# Larval Population Density Alters Adult Sleep in Wild-Type *Drosophila* *melanogaster* but Not in *Amnesiac* Mutant Flies

**DOI:** 10.3390/brainsci4030453

**Published:** 2014-08-11

**Authors:** Michael W. Chi, Leslie C. Griffith, Christopher G. Vecsey

**Affiliations:** 1 National Center for Behavioral Genomics, Brandeis University, Waltham, MA 02454, USA; E-Mails: mchi15@brandeis.edu (M.W.C.); griffith@brandeis.edu (L.C.G.); 2 Volen National Center for Complex Systems, Brandeis University, Waltham, MA 02454, USA; 3 Department of Biology, Brandeis University, Waltham, MA 02454, USA; 4 Department of Biology, Swarthmore College, Swarthmore, PA 19081, USA

**Keywords:** *Drosophila*, sleep, development, social isolation and enrichment, population density, *amnesiac*, *or83b*

## Abstract

Sleep has many important biological functions, but how sleep is regulated remains poorly understood. In humans, social isolation and other stressors early in life can disrupt adult sleep. In fruit flies housed at different population densities during early adulthood, social enrichment was shown to increase subsequent sleep, but it is unknown if population density during early development can also influence adult sleep. To answer this question, we maintained *Drosophila* larvae at a range of population densities throughout larval development, kept them isolated during early adulthood, and then tested their sleep patterns. Our findings reveal that flies that had been isolated as larvae had more fragmented sleep than those that had been raised at higher population densities. This effect was more prominent in females than in males. Larval population density did not affect sleep in female flies that were mutant for *amnesiac*, which has been shown to be required for normal memory consolidation, adult sleep regulation, and brain development. In contrast, larval population density effects on sleep persisted in female flies lacking the olfactory receptor *or83b*, suggesting that olfactory signals are not required for the effects of larval population density on adult sleep. These findings show that population density during early development can alter sleep behavior in adulthood, suggesting that genetic and/or structural changes are induced by this developmental manipulation that persist through metamorphosis.

## 1. Introduction

Sleep is a crucial behavior in a wide range of animals, as evidenced by the widespread mental and physical issues that result from extended sleep loss [1,2,3,4]. Humans spend almost a third of their lives asleep, yet there are many remaining questions about this ubiquitous behavioral state. For example, how is the set point for sleep need determined? Much research has uncovered genes, signaling molecules, and cells that are involved in sleep regulation [5,6,7], and it clear that daily experiences during adulthood influence sleep amounts and patterns. For example, sleep deprivation in multiple species causes subsequent homeostatic increases in sleep density and duration [8,9,10,11]. However, does all inter-individual variation in adult sleep result from differences in current environmental conditions and underlying genetics, or is there a component of developmental experience and plasticity? How do experiences during early development shape our adult sleep behavior? 

To address this issue, we have studied the fruit fly, *Drosophila melanogaster*. Fruit flies are well suited to this area of research because they develop rapidly (10–12 days from fertilization to adult) and are particularly amenable to examination of the genes that control behavior [12,13,14]. Importantly, sleep in fruit flies has many similarities to sleep in mammals at the behavioral, pharmacological, molecular, and electrophysiological levels [10,15,16,17,18,19,20].

Recently, it has been shown that increased population density during early adulthood in flies results in increased sleep during the following days [21,22]. This effect was attributed to the “social enrichment” that resulted from the increase in population density. Enrichment was theorized to cause an increase in learning events, stimulating an increase in sleep in order to improve plasticity mechanisms underlying storage of the newly learned information. However, it is unknown whether changes in conditions during earlier stages of development also play a role in controlling the amount or quality of sleep. Therefore, in this study, we have asked whether alterations in population density during larval development play a role in setting adult sleep patterns.

## 2. Results

### 2.1. Effects of Larval Population Density on Sleep in Wild-Type Drosophila Females

**Figure 1:**
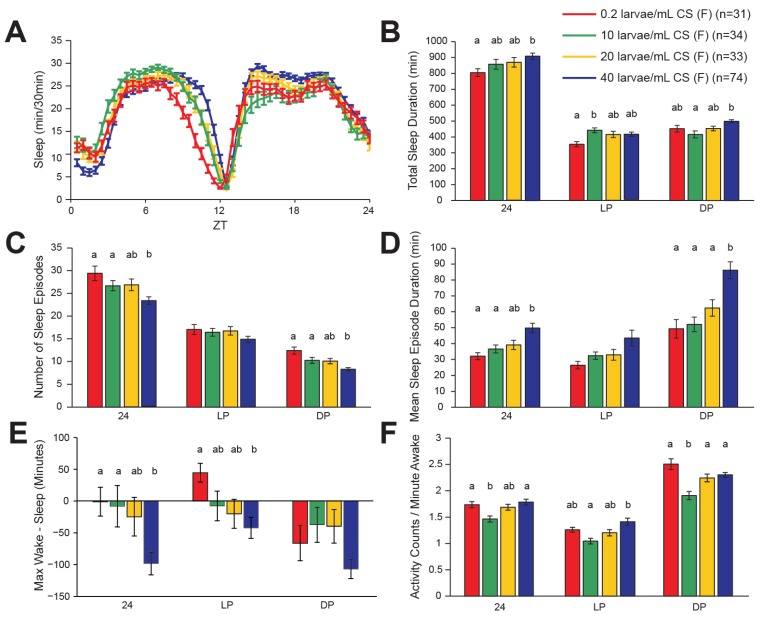
Increasing larval population density causes greater sleep consolidation in adult female fruit flies. (**A**) Minutes of sleep per 30-min bin, across an average day consisting of 12 h of light followed by 12 h of darkness (LD). Time after lights-on is indicated by zeitgeber time (ZT) on the *X*-axis. (**B**–**F**) Quantification of sleep parameters for the same average LD day, across all 24 h, across the light period (LP), or across the dark period (DP). Error bars represent the standard error of the mean. *n* represents the number of flies in each group. Letters above columns are present in cases where a significant effect of population density was observed, and indicate significance groups from *post-hoc* analysis. Columns within each group of four that do not have any letter in common are significantly different from each other. (**B**) Total sleep duration. (**C**) Number of sleep episodes. (**D**) Mean sleep episode duration. (**E**) A metric for relative stability of wake and sleep states, calculated by subtracting the duration of the longest sleep episode from the duration of the longest wake episode. Thus, positive values indicate that wakefulness was more consolidated than sleep, and *vice versa*. (**F**) Total number of beam crosses, or activity counts, per minute of wakefulness. Increasing larval population density had several effects on female sleep characteristics, tending to decrease the number of sleep episodes while increasing their duration, resulting in more consolidated sleep.

**Figure 2:**
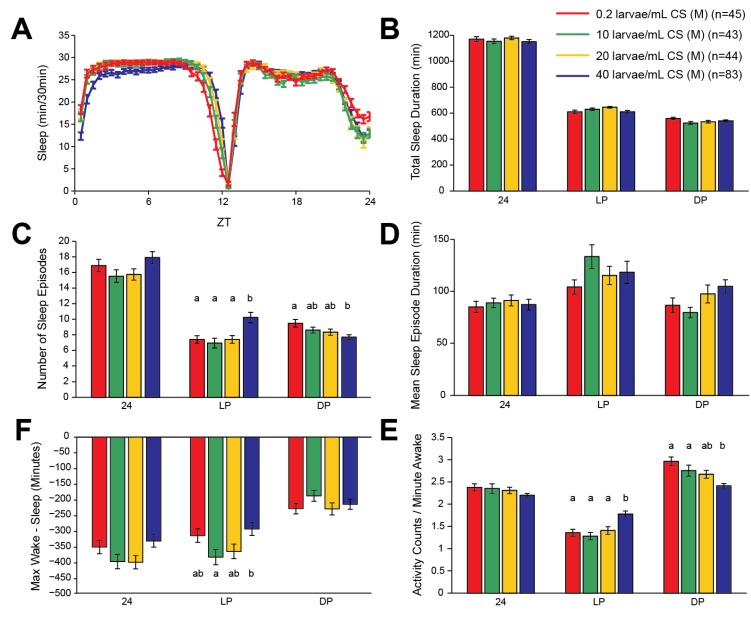
Increasing larval population density has modest effects on sleep characteristics in adult male fruit flies. (**A**) Minutes of sleep per 30-min bin, across an average day consisting of 12 h of light followed by 12 h of darkness (LD). Time after lights-on is indicated by zeitgeber time (ZT) on the *X*-axis. (**B**–**F**) Quantification of sleep parameters for the same average LD day, across all 24 h, across the light period (LP), or across the dark period (DP). Error bars represent the standard error of the mean. n represents the number of flies in each group. Letters above columns are present in cases where a significant effect of population density was observed, and indicate significance groups from *post-hoc* analysis. Columns within each group of four that do not have any letter in common are significantly different from each other. (**B**) Total sleep duration. (**C**) Number of sleep episodes. (**D**) Mean sleep episode duration. (**E**) A metric for relative stability of wake and sleep states, calculated by subtracting the duration of the longest sleep episode from the duration of the longest wake episode. Thus, positive values indicate that wakefulness was more consolidated than sleep, and *vice versa*. (**F**) Total number of beam crosses, or activity counts, per minute of wakefulness. There were no significant effects of larval population density in male flies across the 24-period.

The prevailing method of assessing sleep patterns in flies is to record activity by placing individual flies in small glass tubes, in which an infrared beam crossing the tube at the midline is broken each time the fly passes the midline. Periods of 5 min or more without a single beam break are marked as a sleep bout [23]. Simultaneous video tracking has shown that this beam-break data is quite reliable at detecting periods of quiescence, because awake flies rarely spend 5 min on one side of the tube without crossing the midline [24]. Data were averaged across days 2–6 of exposure to a 12 h/12 h light/dark schedule (LD). Male and female wild-type Canton-S (CS) *Drosophila* exhibited classic crepuscular rest/activity patterns, meaning that they had active periods around dawn (ZT 0) and dusk (ZT 12), with a midday siesta and a more consolidated period of sleep during the dark period. Despite the similarity in pattern, males slept much more than females, especially during the light period (Figure 1A and Figure 2A), consistent with previous reports [8,25]. We calculated several sleep parameters for each sex, including total sleep time, number of sleep episodes, mean duration of sleep episodes, total activity counts, and mean activity counts per minute awake, and these were tabulated over the light period, the dark period, and the full 24 h.

In female flies, there was a significant effect of larval population density on total sleep duration, both during the light period (*F*(3,168) = 3.97, *p* = 0.0091), the dark period (*F*(3,168) = 5.55, *p* = 0.0012), and across the entire day (*F*(3,168) = 2.97, *p* = 0.034). The largest difference was between the isolated 0.2 larvae/mL group and the most densely populated 40 larvae/mL group (Figure 1B). In addition to the change in overall sleep levels, the structure of sleep in female flies was also altered by larval population density. The number of sleep episodes across the entire day was inversely related to population density (*F*(3,168) = 5.75, *p* = 0.0009). This same effect was observed during the dark period alone (*F*(3,168) = 9.84, *p* < 0.0001), with no significant effect during the light period (*F*(3,168) = 1.59, *p* = 0.19) (Figure 1C). This reduction in the number of sleep episodes with increasing population density was paired with an increase in the mean sleep episode duration across the 24-h period (*F*(3,168) = 7.00, *p* = 0.0002). This effect was again most prominent during the dark period (*F*(3,168) = 10.67, *p* < 0.0001), although a marginally significant effect was also observed during the light period (*F*(3,168) = 2.73, *p* = 0.046) (Figure 1D). Thus, in female flies in LD conditions, increasing larval population density produced more consolidated sleep (longer, less frequent episodes). This effect was evident in a separate metric for behavioral state stability, in which the longest sleep episode was subtracted from the longest wake episode (Figure 1E). There was a significant effect of population density on this measure across the entire day (*F*(3,168) = 4.23, *p* = 0.007 and during the light period (*F*(3,168) = 3.30, *p* = 0.02, and a borderline significant effect during the dark period (*F*(3,168) = 2.65, *p* = 0.051. The overall trend was for flies raised at higher larval population densities to have longer sleep episodes relative to wake episodes.

It was possible that the observed increase in sleep with increased larval population density was due to general lethargy or unhealthiness of the flies. To address this question, we calculated the number of activity counts per minute of wakefulness, which serves as a measure of locomotor ability. There were significant overall effects of larval population density on activity while awake, during the light period (*F*(3,168) = 5.57, *p* = 0.0012), the dark period (*F*(3,168) = 10.60, *p* < 0.0001), and averaged across a day in LD (*F*(3,168) = 5.23, *p* = 0.0018). However, greater sleep consolidation did not appear to be caused by reduced locomotion. For example, the groups with the largest disparity in larval population (40 larvae/mL and 0.2 larvae/mL groups) had the largest difference in sleep consolidation (Figure 1C–E), but did not have significantly different levels of activity while awake (Figure 1F).

### 2.2. Effects of Larval Population Density on Sleep in Wild-Type Drosophila Males

Compared with females, the effects of larval population density on sleep patterns were less robust in male flies. There was no significant effect of larval population density on total sleep duration across the light period (*F*(3,211) = 2.11, *p* = 0.10), dark period (*F*(3,211) = 2.15, *p* = 0.09), or the entire 24-h period (*F*(3,211) = 0.61, *p* = 0.61) (Figure 2B). The number of sleep episodes during the dark period was affected in male flies similarly as in female flies, with more episodes with lower larval population densities (*F*(3,211) = 4.01, *p* = 0.0084) (Figure 2C). Specifically, the 0.2 larvae/mL and 40 larvae/mL groups were significantly different. During the light period, there was no discernible trend across population densities, although there was a significant effect (*F*(3,211) = 6.84, *p* = 0.0002) due to more sleep episodes in the 40 larvae/mL group compared with the other groups (this was the opposite of the effect seen in female flies). This meant that, combined across the 24-h period, there was no overall effect of population density on sleep episode number (*F*(3,211) = 2.13, *p* = 0.10). There were no significant effects of larval population density on the duration of sleep episodes during either the light period (*F*(3,211) = 1.08, *p* = 0.36) or across the entire 24-h period (*F*(3,211) = 0.21, *p* = 0.89), and only a marginal effect during the dark period (*F*(3,211) = 2.67, *p* = 0.05) (Figure 2D). As opposed to female flies, in which increasing levels of larval population density clearly favored longer maximum sleep episodes compared with maximum wake episodes, there was no such clear trend in male flies (Figure 1E). This metric was only significantly altered by population density during the light period (*F*(3,211) = 3.52, *p* = 0.016), but not during the dark period (*F*(3,211) = 0.98, *p* = 0.41) or when calculated across the entire day (*F*(3,211) = 2.58, *p* = 0.055).

We also found that activity levels during wakefulness, when averaged across the entire day, were not significantly affected by larval population density in male flies (*F*(3,211) = 1.53, *p* = 0.21). However, this lack of an effect was due to opposing effects during the light (*F*(3,211) = 9.91, *p* < 0.0001) and dark (*F*(3,211) = 8.59, *p* < 0.0001) periods, with the most densely populated 40 larvae/mL group having the highest activity during the light, but the lowest activity during the dark (Figure 2F).

The lack of strong effects of larval population density on sleep patterns in male flies suggests a sex-dependent reliance on developmental conditions for the setting of adult sleep characteristics. Alternatively, the lack of an effect in males may be due to the fact that male flies generally sleep much more than females. This may have resulted in a ceiling effect, such that the dynamic range in sleep levels was so small that no changes could be discerned across different larval population densities. Because males appear less affected by larval population density, in subsequent experiments comparing wild-type and mutant flies, only female flies were tested.

### 2.3. The Effects of Larval Population Density on Sleep in Wild-Type and Amnesiac Mutant Female Flies

It had previously been shown that alterations in larval population density cause structural changes in the adult *Drosophila* brain [26,27,28]. Notably, one of the observed effects was that greater population density (25 larvae/mL compared with 5 larvae/mL) resulted in increased volume of the calyx of the adult mushroom body (MB) [26]. The MB is an important integrative center in the fly brain [29,30,31], and is involved in the regulation of sleep [32,33,34]. Therefore, we hypothesized that the effects of larval population density on adult sleep might be due to structural changes in the MB. Previous research found that mutation of the memory gene *amnesiac* [35,36] eliminated the effect of larval population density on the structure of the MB, whereas mutation of other genes involved in learning and memory (*rutabaga* and *radish*) did not [26]. We therefore asked if *amnesiac* was also required for the effects of larval population density on adult sleep. To test this possibility, we repeated the experiment described in Section 2.1 using CS, *amn^1^*, and *amn^x8^* flies. For this experiment, we focused on females of the 0.2 larvae/mL and 40 larvae/mL population densities, where we had observed the largest differences. Unfortunately, *amn^x8^* larvae in the 0.2 larvae/mL group had extremely poor survival to adulthood, and thus we were unable to assess the effects of larval population density in that line of flies.

**Figure 3:**
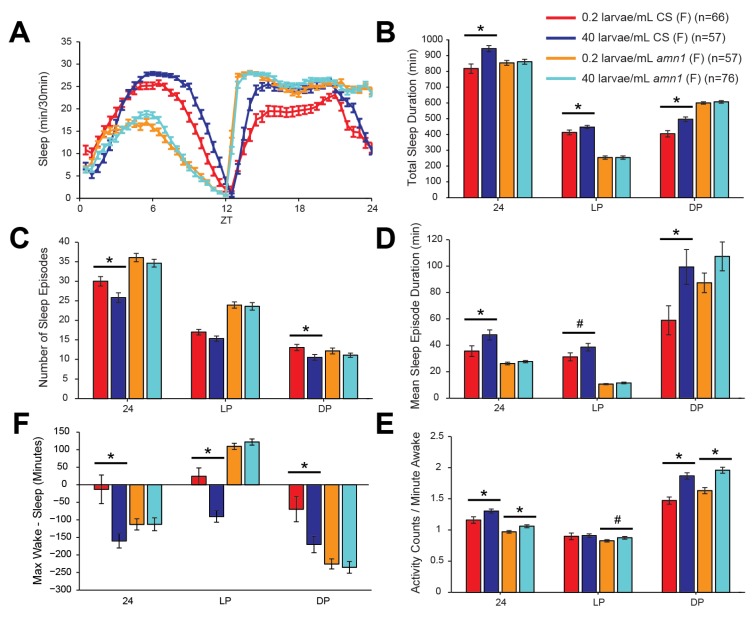
Mutation of the gene *amnesiac* blocked the effects of larval population density on adult sleep in female flies. (**A**) Minutes of sleep per 30-min bin, across an average day consisting of 12 h of light followed by 12 h of darkness (LD). Time after lights-on is indicated by zeitgeber time (ZT) on the *X*-axis. (**B**–**F**) Quantification of sleep parameters for the same average LD day, across all 24 h, across the light period (LP), or across the dark period (DP). Error bars represent the standard error of the mean. *n* represents the number of flies in each group. * Indicates *p* < 0.05 for a comparison of the two bars under the line. # Indicates 0.05< *p* < 0.10. (**B**) Total sleep duration. (**C**) Number of sleep episodes. (**D**) Mean sleep episode duration. (**E**) A metric for relative stability of wake and sleep states, calculated by subtracting the duration of the longest sleep episode from the duration of the longest wake episode. Thus, positive values indicate that wakefulness was more consolidated than sleep, and *vice versa*. (**F**) Total number of beam crosses, or activity counts, per minute of wakefulness. The promotion of sleep consolidation seen with increased larval population density in CS flies was inhibited in *amn^1^* flies. In contrast, larval density was associated with hyperactivity while awake during the dark period, and this effect was not mitigated by mutation to *amnesiac.*

We observed that *amn^1^* flies had quite different sleep patterns from CS flies, similar to previous reports [37]. Although we did not quantify these differences, daytime sleep in *amn^1^* flies was strongly reduced, as was the latency to sleep after lights were turned out at ZT12 (Figure 3A,B). Sleep was also more fragmented in *amn^1^* flies, with increased numbers of shorter sleep bouts during the day (Figure 3C,D). In CS flies, increased larval population density again caused significantly higher levels of total sleep, as well as increased sleep consolidation, with decreased sleep episode number, an increase in mean sleep episode duration, and a shift towards greater sleep state stability (Figure 3A–E), recapitulating the effects observed when comparing the 0.2 larvae/mL and 40 larvae/mL groups in Figure 1. Most interestingly, across all of these sleep characteristics, the effects of larval population density were absent in *amn^1 ^* flies (Figure 3A–E).

We again measured activity per minute awake to determine if increased sleep due to greater larval population density could be explained by lethargy during wakefulness. This metric actually showed an increase in waking activity, especially during the dark period, with greater larval population density (Figure 3F). This significant effect on activity was also observed in *amn^1^* flies, with significant increases across the entire day and during the dark period, and with a trend towards significance during the light period. The fact that mutation to *amnesiac* prevented the effects of larval population density on adult sleep characteristics but had no effect on changes in waking activity suggests that larval density has both *amnesiac*-dependent and *amnesiac*-independent effects on adult behavior.

### 2.4. Effects of Larval Population Density on Sleep Persist in Flies Lacking the Olfactory Receptor or83b

Social interaction in flies has a strong olfactory component, and previous research has shown that social interactions can affect both sleep [21,22] and circadian rhythms [38,39]. To examine if the effects of larval population density on adult sleep behavior were mediated by olfaction, we studied an olfactory mutant (*or83b*) that impairs flies’ responses to a wide range of odors [40]. Interestingly, this mutant has been found to block the ability of flies to synchronize their circadian rhythms in response to social interactions [39]. Additionally, silencing neurons by expressing tetanus toxin under the control of the *or83b* promoter was found to prevent increases in sleep resulting from increased population density during early adulthood [21].

**Figure 4:**
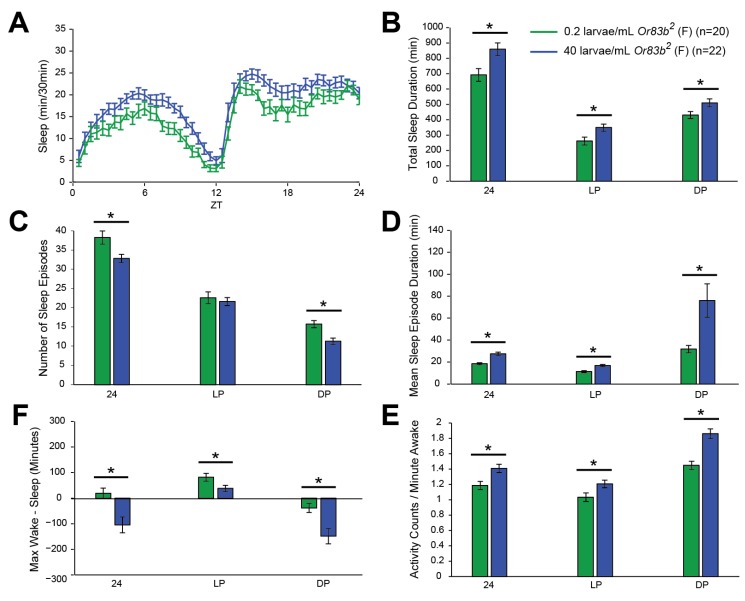
Increasing larval population density causes greater sleep consolidation in adult female fruit flies that lack the olfactory receptor *or83b*. (**A**) Minutes of sleep per 30-min bin, across an average day consisting of 12 h of light followed by 12 h of darkness (LD). Time after lights-on is indicated by zeitgeber time (ZT) on the *X*-axis. (**B**–**F**) Quantification of sleep parameters for the same average LD day, across all 24 h, across the light period (LP), or across the dark period (DP). Error bars represent the standard error of the mean. n represents the number of flies in each group. (**B**) Total sleep duration. (**C**) Number of sleep episodes. (**D**) Mean sleep episode duration. (**E**) A metric for relative stability of wake and sleep states, calculated by subtracting the duration of the longest sleep episode from the duration of the longest wake episode. Thus, positive values indicate that wakefulness was more consolidated than sleep, and *vice versa*. (**F**) Total number of beam crosses, or activity counts, per minute of wakefulness. As in wild-type CS flies, increases in larval population density were associated with greater sleep consolidation during adulthood in *Or83b^2^* flies.

We found that *or83b* mutant flies (*Or83b^2^*) showed a similar pattern of sleep alterations due to larval population density as did wild-type CS flies, with increased total sleep and increased sleep consolidation with greater larval populations (Figure 4). This experiment demonstrates that increased sleep due to larval population density can be observed in another genetic background, emphasizes the specificity of the blockade of this effect in the *amnesiac* mutant, and suggests that the effects of larval population density are not mediated primarily by olfaction. 

## 3. Discussion

Taken together, these findings indicate that increased population density during larval development can promote sleep consolidation in adult *Drosophila* *melanogaster*, with more prominent effects in females than in males, and suggest that the gene *amnesiac* is important for this effect. 

It is interesting to compare our results with studies of sleep in *Drosophila* following social enrichment and isolation during early adulthood [21,22]. In our work, larger populations were consistently associated with increased sleep, resulting from longer sleep bouts. In the studies in which population sizes were manipulated during early adulthood, sleep was also increased, but the most prominent sleep-promoting effect was observed during daytime sleep, whereas in our study both daytime and nighttime sleep were affected. We also observed reductions in the number of sleep episodes with increased population density, but in studies of adult social enrichment [21,22], sleep increases were mediated by an increase in sleep episode duration with no change in sleep episode number. This likely reflects differences in the mechanisms by which larval and early adult social experiences influence sleep. 

What might be the genetic mechanisms that control the responses to larval and adult population density? In our study, the *amnesiac* mutant *amn^1^* did not show changes in sleep due to population density (Figure 3), suggesting that the plasticity-related *amnesiac* gene [35,36,41] is involved. *Amnesiac* has previously been implicated in mediating the effects of larval population density on development of the region of the fly brain called the mushroom body, specifically the size of the calyx subregion [26]. Therefore, it is possible that in response to altered population density, *amnesiac*-dependent remodeling of the mushroom bodies occurs during development, which subsequently affects sleep in adulthood. Since the mushroom bodies have been identified recently as a brain structure that can influence sleep [32,33,34], this is an appealing possibility. It would be of future interest to determine if the volume of the calyx generally correlates with sleep consolidation in adult flies.

In contrast, Ganguly-Fitzgerald and colleagues [21] found that mutations in many genes related to learning, memory, and plasticity blocked the increase in sleep in response to social enrichment, but did not report if *amnesiac* mutants were one of the strains tested that had normal sleep responses. As observed due to increased larval population density [26], social enrichment during early adulthood [28,42] is also associated with structural changes to the mushroom bodies (MBs). The structural effects of adult social enrichment were dependent on cAMP pathway genes such as the cAMP-specific phosphodiesterase *dunce* and the adenylate cyclase *rutabaga* [42]. In contrast, *rutabaga* and *radish* (another memory-related gene [43] that is a putative PKA target [44]) were dispensable for the effects of larval enrichment on MB structure, whereas *amnesiac* was necessary [26]. These combined results suggest that experience during both larval development and early adulthood may influence structural plasticity in the MBs, and thus alter sleep, but may do so through different mechanisms. However, we have not tested if genes that were required for the effects of adult enrichment on sleep, such as *dunce* or *rutabaga* [21], are involved in the effects of larval population density on adult sleep. Therefore, explicit tests of larval development in additional mutant strains will be necessary to show this dissociation conclusively.

Our work therefore raises questions about how *amnesiac* is affected by population density, and how it produces structural changes in the mushroom bodies. *Amnesiac* is thought to code for a PACAP-like neuropeptide [35] that is capable of stimulating cyclic AMP (cAMP) signaling [45], although its mechanism of action is not well understood. Our observed effect persists into adulthood, suggesting either a long-term change in physiology engendered by a short-lived *amnesiac*-dependent event initiated by changes in larval density or perhaps some more permanent change in *amnesiac*-dependent processes. Both would be consistent with structural changes in the brain. Social isolation and related social stressors can produce structural changes in the mammalian brain [46,47,48,49,50,51]. In humans, developmental conditions have prominent effects on adult physiology and behavior [52,53,54,55], including alterations in sleep [56,57]. Thus, similar mechanisms may be induced by altered developmental conditions in mammals as in fruit flies.

While one might expect the effects of larval density on locomotion and sleep to be mechanistically related, our data suggest that they occur via distinct pathways. Mutation of the *amnesiac* gene blocks the action of larval density on sleep, but not its actions on locomotor activity. This suggests that the effects of mutation of *amnesiac* on sleep are not simply a consequence of diminished plasticity in general, as plasticity of other processes is intact. Determining at what points these developmentally triggered cascades interact may give us insight into how the brain builds related but separately controllable behavioral circuits.

In the current study, we focused on the effects of larval population density on adult sleep in standard light/dark conditions. However, it would be of future interest to test flies in constant darkness to allow us to determine if circadian rhythms are affected as well. Social interactions in adult flies have been shown to synchronize circadian activity in an *or83b*-dependent manner [39]. In addition, larval experience, such as exposure to ethanol, has previously been shown to alter adult circadian rhythms [58,59]. These findings support the idea that manipulation of population density during larval development might have important ramifications for adult rhythmicity. Female flies have longer circadian periods than male flies, but males have stronger rhythms than females [60]. It would be of future interest to test if larval conditions play a role in establishing these sex-related differences in circadian rhythms. 

A remaining issue is which aspects of larval population density are relevant for the effects on adult sleep. Do larvae experience social interactions that control development and the establishment of a set point for sleep? If so, there are clearly chemical signals emitted by larvae that are involved in larval interactions [38,61,62,63,64], which could affect development at different larval population densities. Here, we found that the *or83b* gene, which is crucial for responses to a broad range of odorants, is not required for the effects of larval population density on adult sleep. This result suggests that olfaction is not required for this phenomenon, although we cannot rule out a role for other forms of chemical sensation, such as gustation, or other senses that could respond to changes in larval population density. It is also possible that population density has indirect effects on development, by altering the quality of the food larvae are able to consume, the temperature experienced by larvae in the food medium, or some other aspect of the environmental milieu. 

A goal for future research will be to separate the roles of different stages of larval development. *Drosophila* larvae experience three larval instar stages, separated by molts. In the current study, larvae were kept at different population densities for the entirety of larval development, as well as portions of embryonic development and pupal development. It would be interesting to limit the duration of altered population density to specific larval stages, to determine if there is a critical period for the physiological changes that result in altered adult sleep and rhythms [59].

Population densities in normal lab conditions would be closest to our 40 larvae/mL group, meaning that the growth conditions for the isolated 0.2 larvae/mL group in our experiment were the most unusual. In the wild, however, fruit fly larvae may develop in sparser populations. Whichever condition is more naturalistic, our data comparing 0.2, 10, 20, and 40 larvae/mL population densities show that 0.2 and 40 larvae/mL densities had the largest changes in adult behavioral parameters such as sleep consolidation, and that the moderate densities tended to have intermediate phenotypes, suggesting that changes in population density can have graded effects on adult sleep behavior. However, we have not performed mathematical modeling on these data, and thus cannot comment on whether the relationship between population density and adult sleep is linear or follows a different pattern. In addition to its insight into biological mechanisms connecting early development to adult sleep behavior, this work therefore also has important methodological implications for researchers studying sleep in *Drosophila*, demonstrating that it is important to control for population density during larval development, as well as early adulthood [21,22], to ensure that each fly line will be starting from the same baseline levels of sleep.

## 4. Experimental Section

### 4.1. Fly Lines and Maintenance

All adult flies were fed standard cornmeal/dextrose food supplemented with yeast pellets, and were housed in plastic paper-stoppered bottles on a 12 h/12 h light/dark cycle in a 25 °C incubator (Percival Scientific, Inc., Perry, Iowa, USA). Fly lines used were the *Canton-Special* (*CS*) wild-type strain of *Drosophila melanogaster*, *amnesiac* mutants *amn^1^* [35] and *amn^x8^* [65], and the *or83b* mutant *Or83b^2^* [40]. *Amn^1^* flies had been previously backcrossed onto a CS background. Flies were obtained from the Bloomington Drosophila Stock Center and the lab of Scott Waddell.

### 4.2. Egg and Larvae Collection

To collect eggs, an egg-laying chamber was used in conjunction with apple juice plates made according to a published protocol [66]. Apple juice plates were made the day before collecting the eggs. A thin layer of yeast paste, about 3 cm in diameter, was added to each plate. Bottles of adult flies were transferred to an empty bottle and then transferred to the egg-laying chamber with one of the apple juice plates to be incubated overnight at 25 °C.

The next day, one hour after lights-on (ZT 1), the incubation plate and egg-laying chamber were taken out and transferred to another apple juice plate. Flies were allowed to lay eggs for 30 min at a time, before being switched to a new plate. This was repeated with the remaining 3 plates. Eggs were collected under a dissecting microscope with a dental pick, and were transferred to test tubes (17 mm diameter × 70 mm length) containing ~5 mL of yeast-free food. Before the eggs were added, the surface of the food in the each test tube was first scored with a spatula to help larvae gain access to the food. Tubes were then placed into the 25 °C incubator to grow. In the first experiment, using only the CS line, 1, 50, 100, or 200 eggs were added to each test tube, resulting in population densities of 0.2, 10, 20, and 40 larvae/mL. In the second experiment, comparing the CS line with mutant strains, only females at densities of 0.1 and 40 larvae/mL were used.

After the eggs hatched into larvae and the larvae reached later stages of pupation (7–8 days post-eclosion), pupae were individually transferred to separate test tubes with a dental pick. The test tubes containing pupae were put in the 25 °C incubator to further develop. After eclosion, adult flies were allowed to mature individually in test tubes containing ~5 mL of yeast-free food for another 3–4 days before being used for sleep experiments.

### 4.3. Sleep Experiments

Male and female flies from each population density were separated under CO_2_ anesthesia, loaded into glass tubes containing a 5% sucrose/1% agarose food pellet at one end, and placed in *Drosophila* Activity Monitoring (DAM) boards (Trikinetics, Inc., Waltham, MA, USA). Boards were put in a 25°C incubator with a 12 h/12 h light/dark schedule that matched that during development. The light/dark schedule was maintained for 6–7 days (LD period). The first day of LD was not analyzed, due to acclimation of the flies to the new environmental conditions. Thus, days 2–6 of LD were averaged and used for subsequent analysis. Activity was measured by infrared beams crossing the center of each tube. Sleep was defined as 5 min or more with no beam-crosses. MATLAB (MathWorks) was used to calculate and compare sleep characteristics of the different larval density populations using previously published scripts [24,67].

### 4.4. Statistics

All statistical analysis was done using JMP Version 5 (SAS Institute, Inc., Cary, NC, USA). Sleep parameters during the light period and dark period of LD were analyzed separately. For the first experiment comparing only CS flies, male and female flies were separated, and each sleep parameter was then analyzed using a one-way ANOVA with population density as the main factor. Each significant ANOVA was followed by Tukey *post-hoc* tests. For the second experiment comparing CS flies *amn* and *or83b* mutants, the low and high population densities were compared with t-tests within each genotype.

## 5. Conclusions 

In this study, we have shown that population density during larval development has an impact on the sleep behavior of adult flies, causing female flies to sleep longer, and in more consolidated bouts. We also demonstrate that these effects are attenuated in flies with a mutation in the *amnesiac* gene, but are not affected by mutation to the ubiquitous olfactory receptor *or83b*. Because *amnesiac* has been implicated in mediating structural plasticity in the mushroom bodies resulting from changes in larval density, this suggests that *amnesiac*-induced changes to this sleep-regulatory brain region could be responsible for the effects we have observed on adult sleep. In general, our findings suggest that developmental conditions are crucial for the establishment of normal sleep patterns in adulthood. It will be of future interest to determine which physiological sequelae of altered larval population density are important for the effects on sleep, which stages of larval development are important for development of adult sleep patterns, and how *amnesiac* is affected by changes in population density.
